# Exploration of immune response mechanisms in cadmium and copper co-exposed juvenile golden cuttlefish (*Sepia esculenta*) based on transcriptome profiling

**DOI:** 10.3389/fimmu.2022.963931

**Published:** 2022-09-23

**Authors:** Xiaokai Bao, Weijun Wang, Xipan Chen, Yanwei Feng, Xiaohui Xu, Guohua Sun, Bin Li, Xiumei Liu, Zan Li, Jianmin Yang

**Affiliations:** ^1^ School of Agriculture, Ludong University, Yantai, China; ^2^ College of Life Sciences, Yantai University, Yantai, China

**Keywords:** Cd and Cu co-exposure, heavy metals, immunity, protein-protein interaction network, *Sepia esculenta*, transcriptome

## Abstract

*Sepia esculenta* is a popular economic cephalopod with high yield, delicious meat, and rich nutrition. With the rapid development of heavy industry and medical industry, a large amount of waste has been released into the ocean recklessly in recent years, inducing a significant increase in the content of heavy metals, especially cadmium (Cd) and copper (Cu), in the ocean. This phenomenon significantly affects the growth and development of *S. esculenta*, causing a serious blow to its artificial breeding. In this study, transcriptome analysis is used to initially explore immune response mechanisms of Cd and Cu co-exposed juvenile *S. esculenta*. The results show that 1,088 differentially expressed genes (DEGs) are identified. And DEGs functional enrichment analysis results suggests that co-exposure may promote inflammatory and innate immune responses in juvenile *S. esculenta*. Fifteen key genes that might regulate the immunity of *S. esculenta* are identified using protein-protein interaction (PPI) network and KEGG enrichment analyses, of which the three genes with the highest number of interactions or involve in more KEGG pathways are identified as hub genes that might significantly affect the immune response processes. Comprehensive analysis of PPI network and KEGG signaling pathway is used for the first time to explore co-exposed *S. esculenta* juvenile immune response processes. Our results preliminarily reveal immune response mechanisms of cephalopods exposed to heavy metals and provide a valuable resource for further understanding of mollusk immunity.

## 1 Introduction

Heavy metals were metallic elements which were difficult to be degraded and easy to accumulate in organisms (1, 2). Due to high toxicity, strong persistence, wide sources, and strong destructive of heavy metals, heavy metal pollution, especially in the ocean, has attracted attention of various countries (3, 4). In recent years, more and more effluent and waste were discharged into oceans with the rapid development of petrochemical industry, fishery, medical industry, and agriculture, resulting in the continuous increase of heavy metal content, especially Cu and Cd, in nearshore oceans, inducing a large amount of heavy metals to accumulate in marine organisms, whether they are caught or cultured, and ultimately have a certain impact on human health (5–8). Organisms living in oceans with heavy metals would accumulate large amounts of heavy metals, which significantly affected the growth, immunity, metabolism, and other processes (4, 9–11). For instance, Sfakianakis et al. found that heavy metal accumulation would induce fish deformities, which had a devastating impact on the growth and survival of fish (12). And Ivanina et al. indicated that heavy metal exposure significantly affected the immune, inflammation, metabolic, and oxidation processes of oyster (13). Cadmium (Cd) and copper (Cu) were toxic heavy metals found in the world’s oceans. They could accumulate in aquatic organisms and could significantly affect the growth, development, movement, and reproduction of them after certain accumulation levels (12, 14–19). For cephalopods, previous studies have shown that Cu can induce octopus tissue peroxidation to induce oxidative damage (20); and Cd was found to accumulate abundantly in digestive glands and induce toxic responses (21). Meanwhile, the immune responses of organisms exposed to Cd or Cu alone were significantly affected. Xie and Wen et al. found that Cd exposure suppressed expressions of immune-related genes in fish, thereby inhibiting the immune response processes (22, 23). And Sheir et al. indicated that Cd could inhibit the innate immune response of shellfish (24). Meanwhile, Cu has been reported in previous research to significantly inhibit the functions of immune cells, and inhibit lysozyme activity and phagocytosis, resulting in immune system disorders (25–27). Hitherto, the effects of Cd and Cu co-exposure on immune response mechanisms have been rarely studied in marine organisms and have not been studied in cephalopods.

Golden cuttlefish (*Sepia esculenta*) was an important economic cephalopod in the world, mainly distributed in the surrounding waters of eastern China, South Korea and the Philippines (28–31). It was loved by people because of its rich nutrition and valuable medicinal value (31, 32). Because of overfishing and harsh ocean conditions, the number of *S. esculenta* wild stocks has declined sharply, and it would even become an endangered species in recent years (33, 34). Under natural conditions, juvenile *S. esculenta* was spawned and hatched in the shallow water near the coast. Juveniles were vulnerable to heavy metal stress, which significantly affected their biological processes and reduced their hatchability and survival rates (35). Therefore, in order to ensure that wild resources would not be exhausted, it was urgent to study the effects of heavy metals on *S. esculenta* juvenile biological processes. Previous studies have shown that Cd and Cu inhibited the growth and development of mollusks and affected their immune response processes (24–26). However, immune response mechanisms of co-exposure on *S. esculenta* have not been widely studied and needed to be further explored.

High-throughput RNA-sequencing (RNA-Seq) was a significant technique for exploring differences between samples at the molecular level. And it was used to study the molecular functions of organisms exposed to heavy metals in recent years. For instance, Zhou et al. found that the processes of endoplasmic reticulum stress, protein modification and apoptosis of *Pocillopora damicornis* changed significantly after Cd exposure through transcriptome (36). And Zhao et al. indicated that Cu exposure induced the oxidative stress in the testis of *Procambarus clarkii* (37). Similarly, it could also be used to explore how Cd and Cu affected the immune response processes of *S. esculenta* at the molecular level.

In our research, we carried out transcriptome sequencing of primary hatching juvenile *S. esculenta* with 24 h Cd and Cu co-exposure. GO, KEGG, and protein-protein interaction (PPI) network analyses based on DEGs were used to explore key genes and pathways affecting juvenile biological functions. We first studied *S. esculenta* juvenile immune response mechanisms after co-exposure based on comprehensive analysis of KEGG and PPI network. Our results laid a function for exploring the effects of heavy metal pollution on biological processes of cephalopods, and further deepen researchers’ understanding of changes in immune response processes of mollusks after environmental stress.

## 2 Materials and methods

### 2.1 *S. esculenta* breeding and sample collection

*Sepia esculenta* parents in spawning period were caught in the Yellow Sea in mid-July. After a short transport, *S. esculenta* were temporarily reared in a breeding pond with temperature of 21 ± 1°C and salinity of 30.6 ± 0.2 to adapt to the environment. In the meantime, frozen shrimps were used to feed *S. esculenta* three times a day. *Sepia esculenta* laid eggs after a week. These eggs were collected and temporarily reared in perforated plastic pots (dissolved oxygen: 5.6 mg/L) that floated on the surface of flowing water (pH: 8.2; temperature: 20.7 ± 0.6°C; salinity: 30.5 ± 0.2) until hatching. The eggs hatched after 29 days. Two hundred primary hatching juvenile *S. esculenta* (38) were collected equally in two 100 L breeding barrels, respectively. Among these, one hundred juveniles were grown in suitable seawater for 24 h (C), and another 100 juveniles were exposed for 24 h to seawater with both Cd and Cu concentrations of 50 ug/L (CuCd). Finally, these juveniles were collected at 0, 4, and 24 h, respectively, and stored at -80°C.

### 2.2 RNA preparation, library construction, and RNA-Seq

In each group, nine juveniles at each time points were randomly selected, and their RNA were extracted using TRIzol method: normal growth for 0 h (C_0h), normal growth for 4 h (C_4h), normal growth for 24 h (C_24h), Cd and Cu co-exposure for 4 h (CuCd_4h), and Cd and Cu co-exposure for 24 h (CuCd_24h). At each time points, equal molar mass of RNA from three juveniles were pooled as the first replicate for RNA-Seq library construction; and another six juveniles were pooled as the second and the third replicates. The remaining juvenile RNA was used for quantitative RT-PCR (qRT-PCR) verification.

In our research, we constructed library using NEBNext^®^ Ultra™ RNA Library Prep Kit for Illumina^®^. First, we purified mRNA from total RNA using poly-T oligo-attached magnetic beads. Secondly, mRNA broke at high temperatures, and first-strand cDNA was synthesized using the M-MuLV Reverse Transcriptase (RNase H-) based on a template of fragment mRNA and primers of random oligonucleotides. Then, second-strand cDNA was synthesized from dNTPs using DNA polymerase I and RNase H. After purification, double-stranded cDNA was end joining repaired, spliced with poly-A, and connected to the sequencing adaptor. cDNA fragments of preferentially 250~300 bp in length were selected using AMPure XP system (Beckman Coulter, Beverly, USA), and PCR was used to amplified these fragments. In the end, PCR products were purified (AMPure XP system), and the library quality was assessed on the Agilent Bioanalyzer 2100 system. Juvenile *S. esculenta* were sequenced by Illumina NovaSeq 6000 (Illumina, USA).

### 2.3 Data quality control, mapping, and differential expression analysis

First, some raw reads were removed including adapter reads, reads containing more than 10% of undetermined bases, and low-quality reads. Then, HISAT2 software (39) was used to map clean reads to reference *S. esculenta* genome (unpublished). And these mapped genes were compared to the NR, NT, SwissProt, KO, KOG, and Pfam databases to find their functions and used for functional enrichment analyses. FPKM was used to analyze expression level and abundance of genes. Finally, DESeq2 was used to identify differentially expressed genes (DEGs) with the criteria p-value ≤ 0.05 and fold change ≥ 1.5 (40). And the union of DEGs at each time points was used for functional analyses.

### 2.4 Enrichment analyses and PPI network analyses

DAVID v6.8 was used to enrich DEGs into the GO terms and KEGG signaling pathways (41). All annotated genes were used as the background gene set, and DEGs were used as a validation set to analyze the functional differences between the control and exposed groups. Then, DEGs were enriched into KEGG pathways and GO terms of biological process, molecular function, and cellular component. Finally, significantly enriched terms and pathways were identified to explore *S. esculenta* juvenile immune response mechanisms after co-exposure. And STRING v11.5 with default parameters was used to construct protein-protein interaction networks using DEGs enriched in the screened significant KEGG signaling pathways (42).

### 2.5 Quantitative RT-PCR assay

Gene-specific primers were designed using Primer Premier 5.0 (**Table 1**) (43). Three reference genes were identified the stability in *S. esculenta* tissues and embryo development stages. And *β*-actin was selected based on its more stable expression level. The specific method of qRT-PCR was described by Li et al. (44).

**Table 1:** List of primers used for qRT-PCR validation.

Gene name	Forward primer (5’-3’)	TM (°C)	Reverse primer (5’-3’)	TM (°C)	Amplicon length (bp)
*NOTCH3*	CCAGACAGCAATGGTGAATA	60	GGTTCCCATTTGTGGAGTT	60	104
*PRKAA1*	CGGGAAGCTGAAGGATAATG	60	CATTGAGCCGGTGATCTAAT	60	134
*ITGA4*	CTCAGACAGGCAAACTTGT	60	CCAGAAAGTCACGGTGTATC	60	128
*DUSP1*	ACGAGTCGGTCATTCTCTAT	60	GACGTGTAACCACCACTTAG	60	142
*TRAF6*	TGACAACACGCTCTTTCTC	60	CGCAACCTTCCTCTCTATTT	60	107
*COL6A3*	CATGTGAGAGTCGGTGTTATC	60	AGTGCTCGGCTTGTATAATG	60	143
*PPP3CA*	GCAAACCAAAGCCAGATG	59	TTCTTGCCGAAGCAATG	58	100
*TNXB*	GCAGCCAACGAGGTTATT	60	GCTCAGCTATGCTACAGTTC	60	123
*COL6A6*	CAACACACTCCTTCCATACAC	61	ACGGGTCCTTGTCCATTA	60	110
*EIF4EBP1*	AAGCTTTCTGCTCCAGTG	60	GACGATAGTCACAAGGGATG	60	116
*LAMC1*	GACAAGGGAATTGGACCTG	60	AACGTGTGTCGTCATTCTC	60	105
*DUSP7*	GGGCAATTACTGGACTTTGA	60	CCAGAAGACAATGGTTGGATAG	61	131
*COL6A4*	GTGTGCTACCACCACTAAAT	60	GGTTTGCTGTCTCCCATATC	60	132
*ATF6B*	GGTTCCAGTGCTGAATACAT	60	ATCTGCTGGGCTTTGAATAG	60	111
*CACNA1D*	CATTGACTGCACCTCCTAAG	60	CTAGCTGGAGCAACCTTTAC	60	151

## 3 Results

### 3.1 Transcriptome sequencing and mapping

Healthy and Cd and Cu co-exposed juvenile *S. esculenta* was sequenced using RNA-Seq method. A total of 669,380,246 raw reads were generated from juvenile *S. esculenta*, and 660,384,274 (98.66%) clean reads were identified after removing reads that contained adapters, low quality reads, and reads with more than 10% uncertain bases (**Table 2**). On average, the Q20 of clean reads was 97.42%, the Q30 of clean reads was 92.99%, and the GC of clean reads was 39.63%. An average of 87.73% clean reads were mapped to our reference genome.

**Table 2:** Sequencing quality and mapping results.

Samples	Raw reads	Clean reads	Q20 (%)	Q30 (%)	GC (%)	Mapping rate (%)
C_0h_1	44,822,088	44,401,358	97.42	93.02	39.89	87.71
C_0h_2	46,604,268	46,067,346	97.39	92.97	38.64	87.25
C_0h_3	42,199,716	41,745,596	97.08	92.31	39.10	86.13
C_4h_1	42,594,570	42,050,900	97.56	93.35	39.79	88.28
C_4h_2	45,122,216	44,583,624	97.37	92.89	40.01	87.80
C_4h_3	43,910,186	43,339,204	97.44	93.00	39.72	87.67
CuCd_4h_1	44,653,518	44,229,734	97.28	92.63	39.89	87.90
CuCd_4h_2	45,007,770	44,566,312	97.65	93.41	39.23	87.93
CuCd_4h_3	45,431,358	44,607,378	97.58	93.30	39.99	88.12
C_24h_1	45,374,672	44,918,056	97.51	93.17	39.82	88.32
C_24h_2	40,894,638	40,402,580	97.59	93.30	38.92	88.10
C_24h_3	42,664,646	42,060,564	97.65	93.45	38.74	87.88
CuCd_24h_1	44,072,002	43,522,418	97.31	92.83	39.85	87.85
CuCd_24h_2	45,068,272	44,699,502	97.54	93.25	38.75	87.31
CuCd_24h_3	44,425,324	43,758,176	97.21	92.48	39.71	87.75

### 3.2 DEGs expression

**Figure 1:**
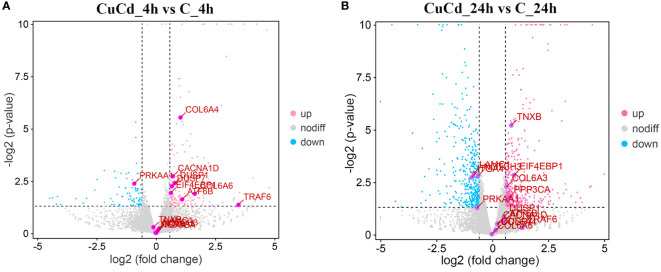
DEGs expression distributions. **(A)** DEGs expression distributions between CuCd_4h and C_4h. Up-regulated DEGs are represented as pink dots; down-regulated DEGs are expressed as blue dots; and non-regulated DEGs are indicated by grey dots. **(B)** DEGs expression distributions between CuCd_24h and C_24h.

**Figure 2:**
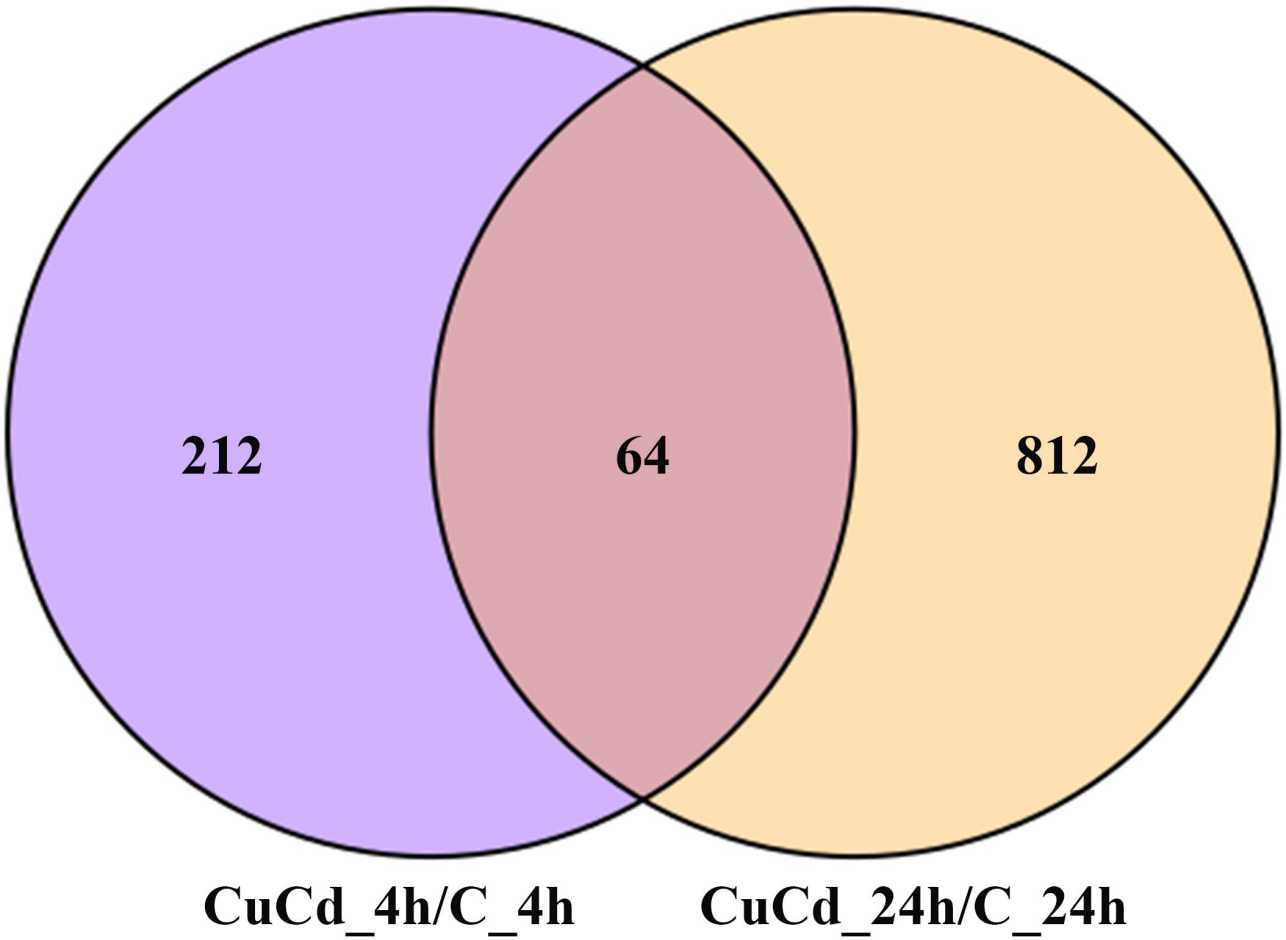
DEGs distributions between two time point. 1,088 DEGs are differential expressed at 4 and 24 h after exposure. 212 DEGs are differential expressed only at 4 h; 812 DEGs are differential expressed only at 24 h; 64 DEGs are differential expressed at both 4 and 24 h.

**Figure 3:**
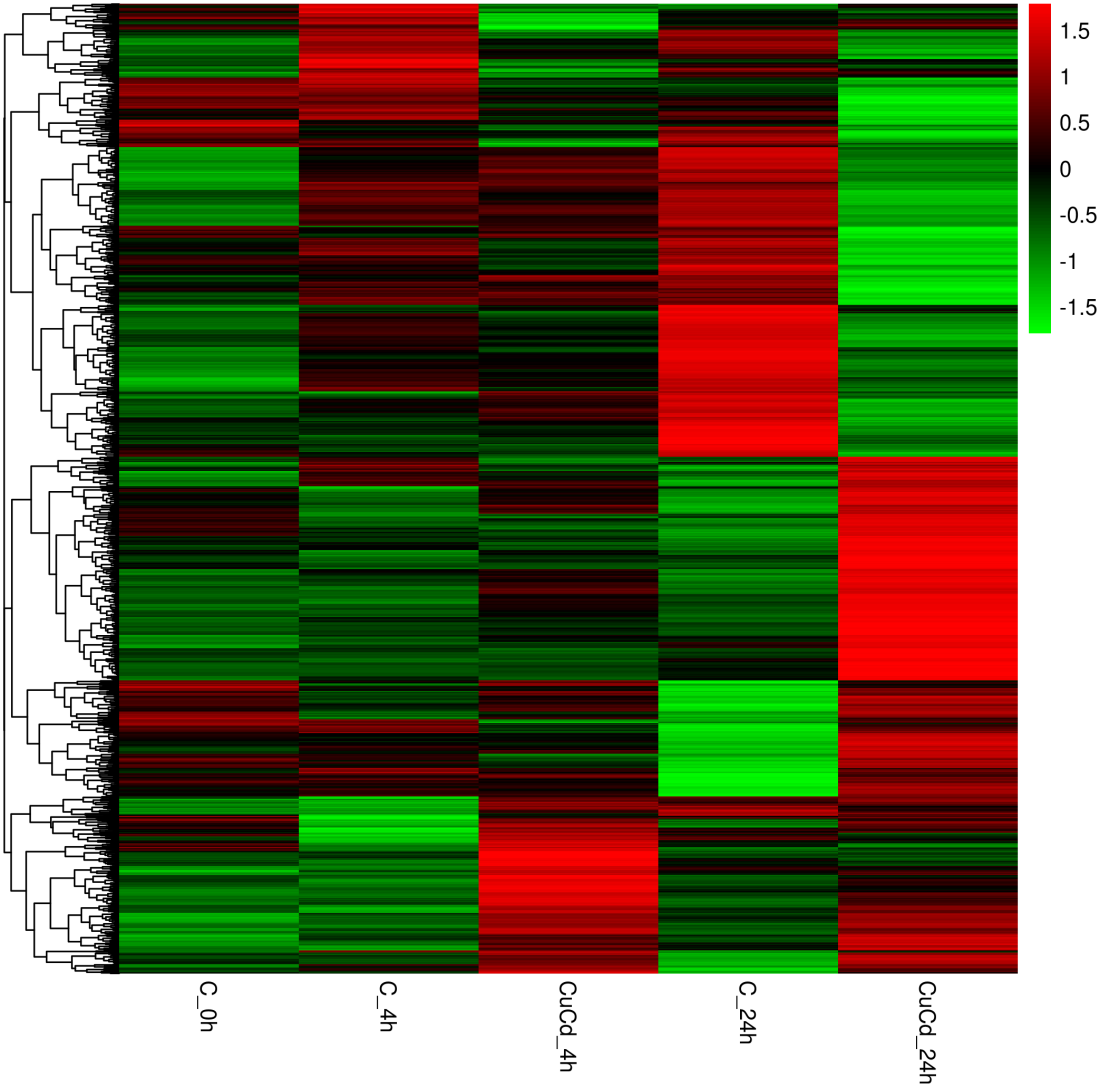
DEGs expression clustering. The gene expressions in different groups are shown in a row; the gene expressions in each group are represented in a column.

Compared with the control group (C), 276 (188 up-regulated and 88 down-regulated) and 876 (441 up-regulated and 435 down-regulated) DEGs were identified at 4 and 24 h, respectively, after co-exposure (**Figure 1**). Venn diagram showed that a total of 1,088 DEGs were differentially expressed, of which 64 DEGs were differentially expressed at both 4 and 24 h (**Figure 2**). And DEGs clustering distribution were visualized in a heatmap (**Figure 3**).

### 3.3 Functional enrichment analyses of DEGs

**Figure 4:**
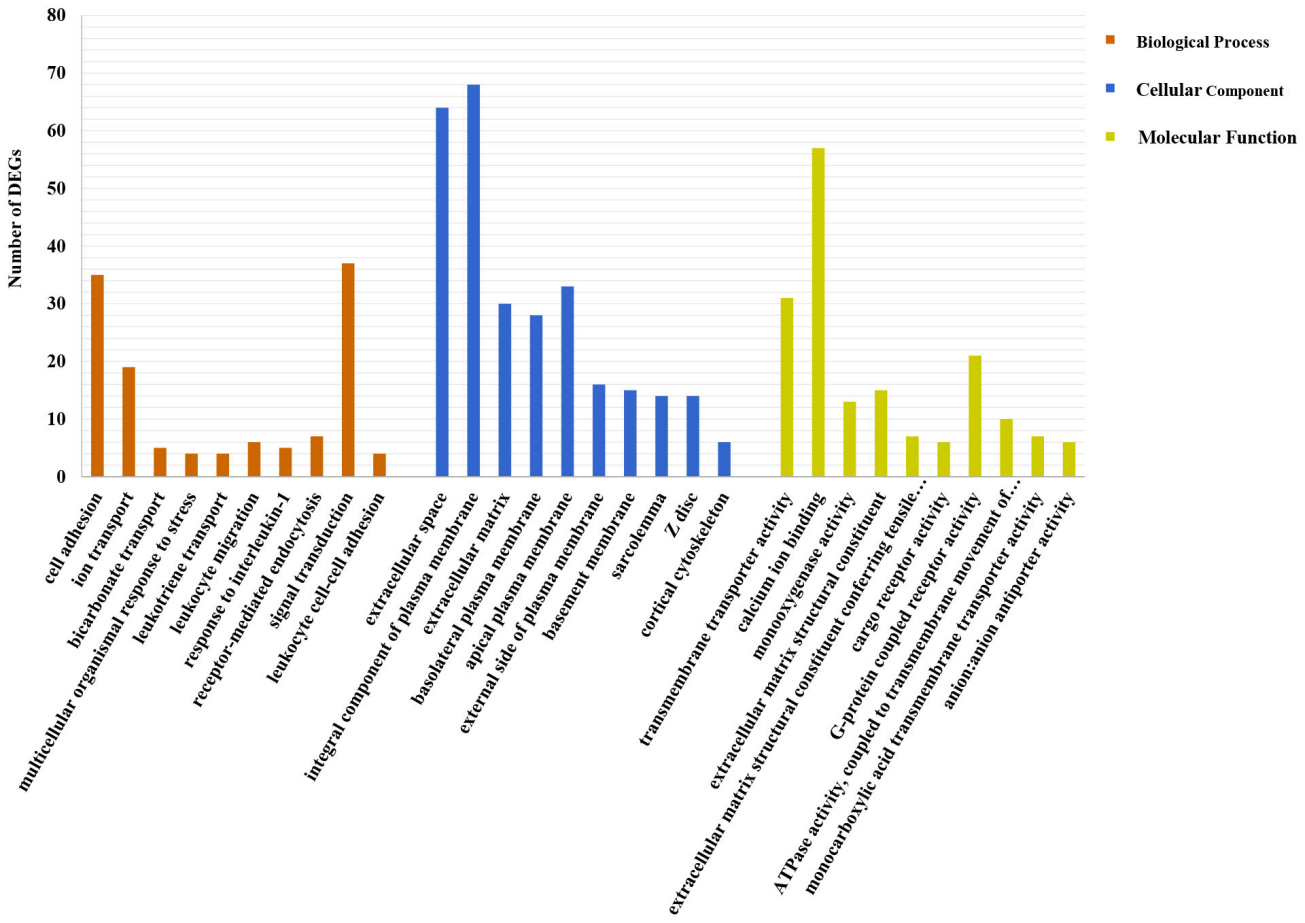
Top 10 significantly enriched GO terms in each cluster. The number of DEGs is shown as the ordinate; specific names of terms are displayed as the abscissa.

**Figure 5:**
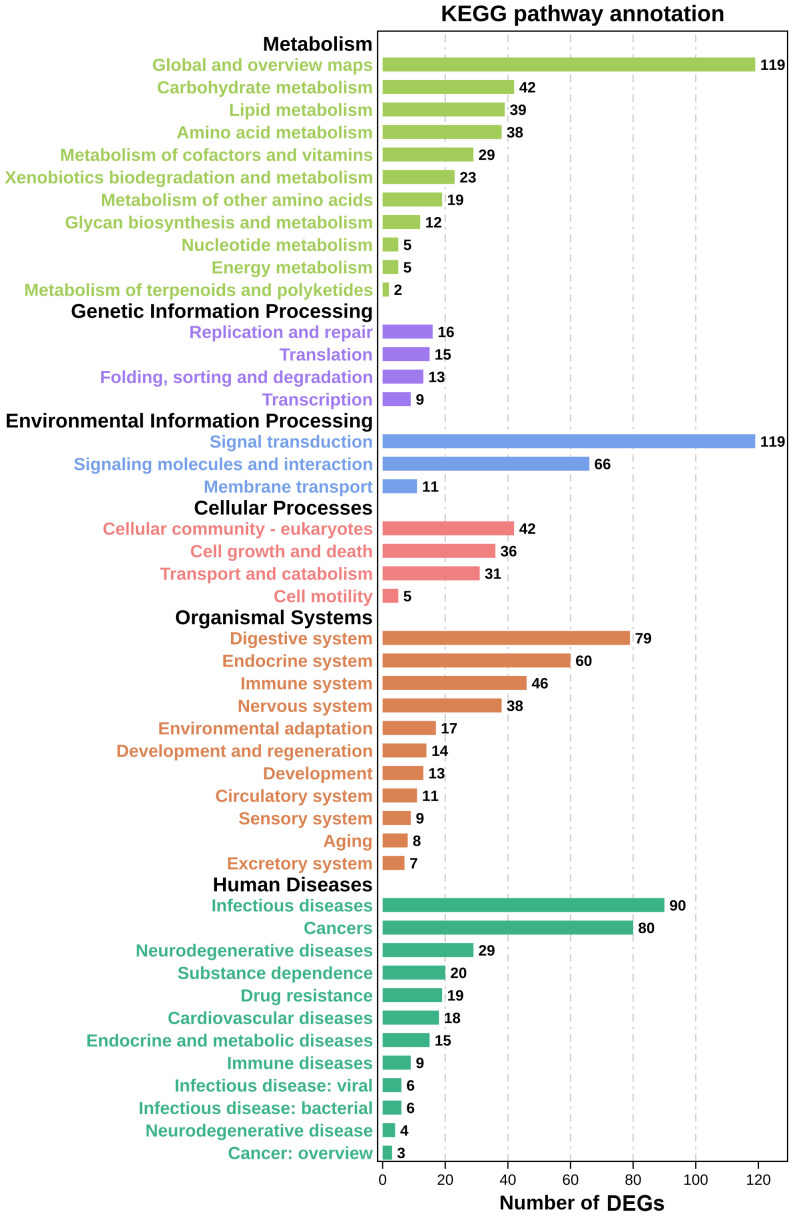
Level-2 KEGG signaling pathways results. The abscissa represents the number of DEGs in the pathway; the ordinate indicates the specific name of these pathways.

DEG functions were analyzed using GO and KEGG enrichment analyses. A total of 137 GO terms (p-value ≤ 0.05) containing three clusters (biological process, cellular components, and molecular function) were significantly enriched in our study. And the top 10 terms in each cluster are shown in **Figure 4**. Among these GO terms, response to interleukin-1, leukocyte migration, and cell adhesion terms in biological process cluster suggested that Cd and Cu co-exposure might induce a series of immune defense processes. The KEGG enrichment analysis results indicated that multiple DEGs were enriched to immune-related level-2 KEGG signaling pathways, including immune system, infectious diseases, and immune disease (**Figure 5**). And nine immune-related KEGG signaling pathways were significantly enriched (**Table 3**). Among them, the enrichment of immune-related pathways such as PI3K-Akt signaling pathway and MAPK signaling pathway suggested that multiple immune cells were activated, and key immune-related genes might be highly expressed after co-exposure.

**Table 3:** Summary of significantly enriched immune-related KEGG pathways after co-exposure.

KEGG pathways	Number of DEGs
Chemical carcinogenesis - DNA adducts	3
Chemical carcinogenesis - receptor activation	8
Herpes simplex virus 1 infection	12
Human papillomavirus infection	9
MAPK signaling pathway	5
MicroRNAs in cancer	3
Natural killer cell mediated cytotoxicity	2
Pathways in cancer	4
PI3K-Akt signaling pathway	11

### 3.4 Screening and validation of key and hub DEGs

**Figure 6:**
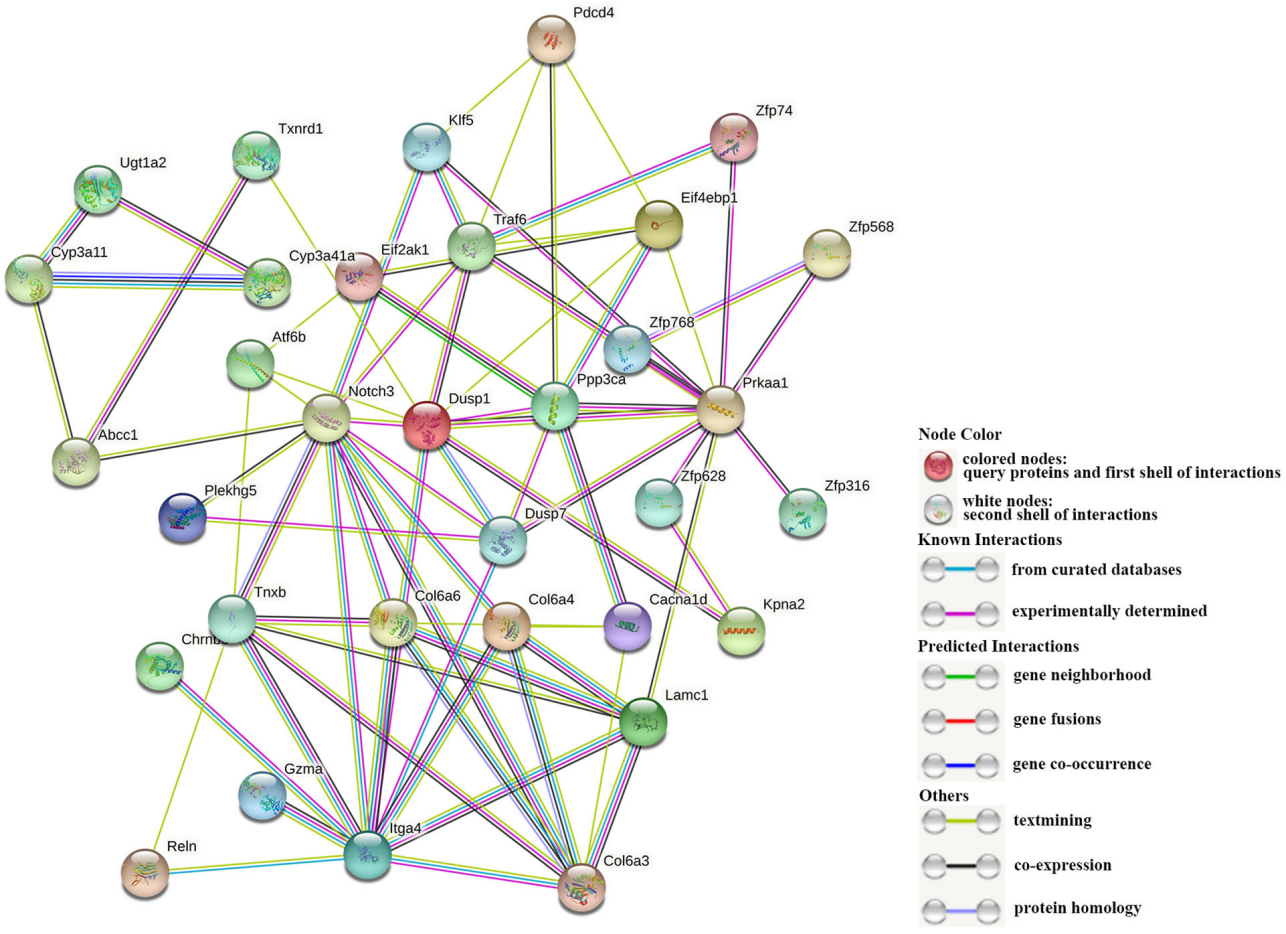
The interaction of DEGs. Each dot represents a protein, and their names are shown next to them. The different lines between dots represent different interactions.

A total of 38 DEGs enriched in KEGG signaling pathways in **Table 3** were used to construct a PPI network (**Figure 6**). And the network parameters are shown in **Table 4**. Above genes have significant interactions, and the average node degree was 4.55. The network clustering coefficient was 0.547, and the p-value was ≤ 8.44E-4. Fifteen DEGs with higher interaction numbers or higher KEGG signaling pathway participation numbers were identified and listed in **Table 5**. Among these genes, NOTCH3, PRKAA1, and ITGA4 interacted with more genes, which were identified as hub genes; and other 12 DEGs with high interaction numbers were defined as key genes. Previous research had shown that these genes were involved in the activation of immune cells and immune signaling pathways and regulated expressions of immune genes. Analysis of above gene functions in juvenile *S. esculenta* would help to further understand immune response mechanisms of *S. esculenta*.

**Table 4:** Summary of immune-related PPI network.

Network statistics	
Number of nodes	33
Number of edges	75
Average node degree	4.55
Clustering coefficient	0.547
Expected number of edges	51
PPI enrichment *p*-value	8.44E-4

**Table 5:** Summary of immune-related key DEGs.

Gene name (abbreviation)	Gene name (official full name)	Number of protein-protein interactions	Number of KEGG signaling pathways
*NOTCH3*	notch receptor 3	12	2
*PRKAA1*	protein kinase AMP-activated catalytic subunit alpha 1	12	1
*ITGA4*	integrin subunit alpha 4	11	2
*DUSP1*	dual specificity phosphatase 1	10	1
*TRAF6*	TNF receptor associated factor 6	7	3
*COL6A3*	collagen type VI alpha 3 chain	7	2
*PPP3CA*	protein phosphatase 3 catalytic subunit alpha	7	2
*TNXB*	tenascin XB	7	2
*COL6A6*	collagen type VI alpha 6 chain	6	3
*EIF4EBP1*	eukaryotic translation initiation factor 4E binding protein 1	6	3
*LAMC1*	laminin subunit gamma 1	6	2
*DUSP7*	dual specificity phosphatase 7	6	1
*COL6A4*	collagen type VI alpha 3 chai4	5	2
*ATF6B*	activating transcription factor 6 beta	4	2
*CACNA1D*	calcium voltage-gated channel subunit alpha1 D	4	1

**Figure 7:**
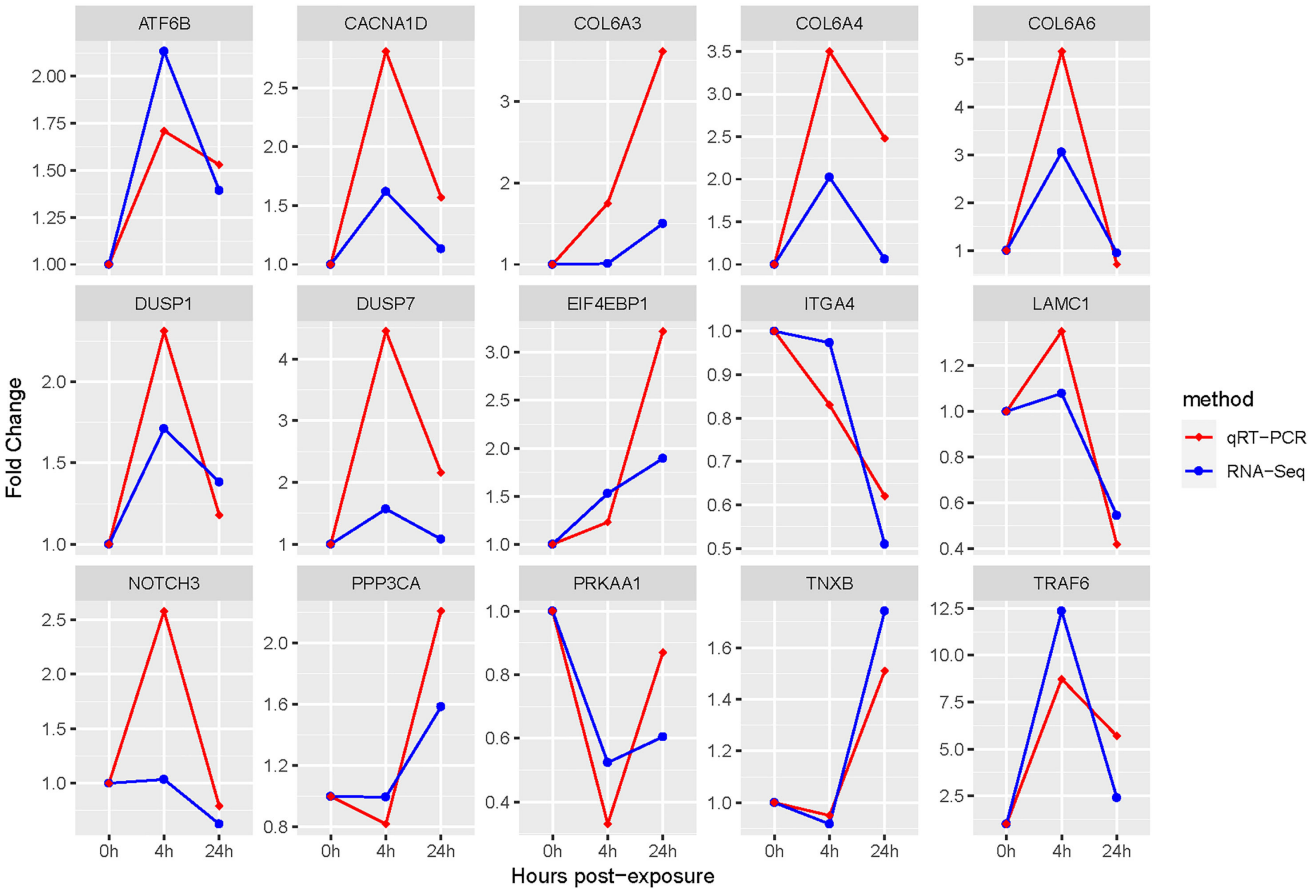
qRT-PCR and RNA-Seq results of key DEGs. The *β-actin* gene is used to normalize the gene expressions of qRT-PCR and RNA-Seq. The abscissa stands for the exposed time of Cd and Cu; the ordinate indicates fold change.

The qRT-PCR was used to verify the accuracy of above gene expressions. The results showed that all DEGs measured were single products. And compared to gene expression profiles of RNA-Seq results, qRT-PCR results showed the same trend, suggesting that the qRT-PCR results were accurate (**Figure 7**).

## 4 Discussion

### 4.1 Cd and Cu-induced immune response in mollusk

As common and high concentrations of heavy metals in the ocean, Cd and Cu could easily accumulate in mollusks and induce immune response processes (24, 45, 46). The effects of Cd and Cu exposure on immunity of shellfish have been studied extensively in the past 10 years. For instance, Cd could suppress immune responses in *Tegillarca granosa* by regulating Ca2+ transport and Ca2+-related apoptosis processes (47). Meanwhile, it could induce immune cell damage in *Perna canaliculus* and destroy the immune system, thereby reducing disease resistance (48). Cu could inhibit the apoptosis, phagocytosis, and adhesion functions of hemocytes, thereby reducing the immune resistance of *Crassostrea rivularis* and *Crassostrea virginica* (49, 50). In addition, Cu exposure has been reported in previous research to inhibit expressions of TLRs and NLRs and inhibit innate immune responses in *Mizuhopecten yessoensis* (51). Cd and Cu-induced immune responses have not been widely studied in cephalopods. This study was designed to preliminarily explore immune response mechanisms of juvenile *S. esculenta* after co-exposure at the molecular level, and laid a foundation for further study cephalopod immunity.

### 4.2 Analyses of DEGs expression

DEGs expression analyses indicated that multiple genes were differently expressed after Cd and Cu co-exposure. The volcano plot results implied that more immune-related genes might be differentially up-regulated with increasing co-exposed time. The Venn diagram analysis revealed that 64 DEGs containing PDCD4, ETV7, and ARG2 were significantly differentially expressed at both 4 and 24 h. These genes have been reported in previous studies to regulate the activation of immune cells and induce multiple immune response processes including inflammatory responses (52–55). We speculated that these might be key genes regulating juvenile immunity after co-exposure, and their specific immune functions in *S. esculenta* needed to be further studied. The results of the heatmap indicated that gene expressions were significantly different after co-exposure and might severely affect *S. esculenta* juvenile immune response processes.

### 4.3 Immune-related functional enrichment analyses

GO enrichment analysis results showed that most of top 10 GO terms of biological processes cluster were significantly related to immune response. The enrichment of response to interleukin-1 term indicated that co-exposure might affect the innate immune response of *S. esculenta* by regulating the expression of IL-1 to regulate TLRs and Toll-like receptor signaling pathways. This result was consistent with previous studies in *Biomphalaria glabrata* and *Mytilus coruscus* (56, 57). The enrichment of cell adhesion term suggested that co-exposure might inhibit *S. esculenta* immune cell adhesion and reduce immune cell activity (58, 59). According to the results of GO enrichment analysis, we preliminarily speculated that immune response of co-exposed juvenile *S. esculenta* might be significantly affected.

Nine immune-related KEGG pathways were significantly enriched in our study. These have been reported to regulate the activation, proliferation, and differentiation of immune cells and immune signal transduction in previous studies, indicating that co-exposure might significantly affect the immune response processes of *S. esculenta* (60–62). Among them, three KEGG signaling pathways identified by previous research as most likely to significantly affect the immune response after co-exposure such as pathways in cancer signaling pathway, MAPK signaling pathway, and PI3K-Akt signaling pathway were screened, identified, and analyzed to further explore *S. esculenta* immune response mechanisms after co-exposure.

#### 4.3.1 Pathways in cancer signaling pathway analysis

Cancer was the deadliest disease in recent decades, and there were few cures (63, 64). After being infected with cancer, some cells grew uncontrollably and proliferated malignantly to form tumor cells (65). And tissue bleeding, decreased vitality, loss of appetite, and other adverse phenomena have also appeared one after another (66, 67). At the same time, immune cells such as T cells and NK cells were activated to suppress and destroy tumor cells (68, 69). Previous studies have shown that pathways in cancer signaling pathway activated WNT signaling pathway, toll-like receptor signaling pathway, and other signaling pathways during cancer and expressed HSPs to regulate immune responses (70–72). Based on our results, it was speculated that co-exposure might induce malignant proliferation of some cells and activated immune signals to promote the processes of *S. esculenta* juvenile immune response. However, cancer has only been initially studied in fish and has not been explored in other aquatic organisms, especially mollusks (73–75). Therefore, the exact immune response mechanisms of juvenile *S. esculenta* in response to cancer were unclear and required further study.

#### 4.3.2 MAPK signaling pathway analysis

MAPK signaling pathway was an important immune regulatory signaling pathway for organisms to induce immune defense responses after biotic and abiotic stresses and played a significant role in the innate immunity of mollusks (76, 77). Previous studies have shown that it was involved in various cellular processes such as proliferation, differentiation, and migration of intestinal immune cells and promoted innate immune responses by activating phagocytic immune responses (78–80). Meanwhile, MAPK signaling pathway has also been found to regulate the production of pro-inflammatory factors such as IL-1β, TNF-α, and TFN-γ to promote inflammatory responses (79, 81, 82). In this study, MAPK signaling pathway was significantly enriched after Cd and Cu co-exposure, indicating that co-exposure might induce the proliferation and differentiation of *S. esculenta* immune cells and promoted inflammatory and innate immune response processes. Three key DEGs, including TRAF6, DUSP1, and DUSP7 were enriched in this pathway, and their expression levels were significantly up-regulated after co-exposure. These have been reported to involved in and regulate MAPK, PI3K, NF-κB, and other signaling pathways to regulate immune responses (83–85), suggesting that juvenile *S. esculenta* might defend against heavy metal stress by regulating innate immune signaling pathways.

#### 4.3.3 PI3K-Akt signaling pathway analysis

As everyone knows that the PI3K-Akt signaling pathway is the core pathway that regulates the immune response and plays a significant part in the proliferation, differentiation, and migration of immune cells (86, 87). It regulates the activation of multiple immune signals such as TLR and NF-κB to promote immune response. At the same time, it can promote inflammatory response by regulating the release of inflammatory factors (88–90). In mollusks, the PI3K-Akt signaling pathway involved in and regulated many physiological and pathological processes of innate immunity (91, 92). For instance, it could regulate phagocytosis to promote innate immune response processes (93). And it can regulate and promote the apoptosis and growth of immune cells and induce immune defense responses after environmental stress (94, 95). In our study, multiple immune-related DEGs such as ATF6B and EIF4EBP1 were enriched into PI3K-Akt signaling pathway, and their expression levels were significantly up-regulated after co-exposure. The above results suggested that the PI3K-Akt signaling pathway might play a crucial role in the immune response processes after co-exposure. The immune response mechanisms of the PI3K-Akt signaling pathway in cephalopods has not been studied. We preliminarily speculated that it might regulate the proliferation and differentiation of *S. esculenta* juvenile immune cells and promote the activation of inflammatory responses and immune signaling factors.

### 4.4 Hub genes functional analysis

In this research, a comprehensive analysis of PPI network and KEGG signaling pathway is used to explore immune functions of juvenile *S. esculenta* co-exposed to Cu and Cd. Three DEGs with highest number of protein interactions or highest KEGG signaling pathway participation numbers including NOTCH3, PRKAA1, and ITGA4 were identified as hub genes most likely to regulate juvenile immunity after Cu and Cd co-exposure. NOTCH3 was a key regulator of cellular function and played a significant role in immune and inflammatory responses (96, 97). Previous studies have shown that NOTCH3 involved in and regulated leukocyte migration and adhesion, tumorigenesis, M2 macrophage infiltration, antigen presentation, activation of cytokines and integrins, tissue inflammation, and multiple immune response processes (98–101). And multiple immune signaling pathways such as PI3K-Akt signaling pathway and rap1 signaling pathway were activated and regulated by NOTCH3 (97). AMPK was a key enzyme widely expressed in cells that regulated various physiological functions such as cell proliferation and autophagy, tumorigenesis, and energy homeostasis (102). And it played an important role in innate immune and inflammation responses (103). As a subunit of AMPK, PRKAA1 played a similar role as AMPK. It promoted the expression of immune genes such as TLR4 and TNF-α to regulate innate immune response (104, 105). Meanwhile, PRKAA1 activated TAK1 and NF-κB to induce inflammatory responses (106). Integrins were an important family of cellular receptors that regulated cell growth, survival, and migration (107, 108). They promoted immune responses primarily by mediating cell adhesion processes (109). ITGA4 was an important integrin subunit efficiently regulating leukocyte adhesion and migration in blood (110). And another function of ITGA4 was recently reported that regulated the infiltration of immune cells such as macrophages, dendritic cells, and neutrophils to induce immune responses (111, 112). In conclusion, we understood that these three genes were closely related to immune and inflammatory responses. However, these have not been studied in cephalopods up to now. An interesting result was that in addition to NOTCH3, which was slightly up-regulated and then down-regulated within 24 h co-exposure, the expression levels of other two genes were continuously down-regulated after co-exposure. Based on above results, we preliminarily speculated that the down-regulation of these genes might inhibit the malignant proliferation of cells to inhibit the generation of tumor cells and prevent excessive inflammatory responses.

### 4.5 Other key DEGs and pathways analyses

In addition to the above three hub genes and three signaling pathways, other identified genes and signaling pathways are also significantly related to immune responses. For instance, COL6A3 is involved in and regulates immune cell infiltration and inflammatory responses (113). And natural killer cell mediated cytotoxicity signaling pathway can regulate the proliferation and apoptosis of NK cells to promote the immune response processes (114, 115). These results further illustrate that co-exposure may significantly affect *S. esculenta* juvenile immunity. Immune response mechanisms of these genes and pathways in co-exposed juvenile *S. esculenta* are unclear until now, and needs to be further studied.

## 5 Conclusion

This study preliminarily explores immune response mechanisms of juvenile *S. esculenta* after Cd and Cu co-exposure using transcriptome analysis. A large number of DEGs suggest that co-exposure may affect juvenile molecular and physiological functions. The results of functional enrichment and PPI network analyses indicate that co-exposure may significantly promote the innate immune and inflammatory responses of *S. esculenta*. In conclusion, co-exposure may significantly affect cuttlefish juvenile immunity, and the results lay a foundation for further understanding of cephalopod immunity after heavy metal exposure.

## Data availability statement

The data presented in the study are deposited in the NCBI repository, accession number SRR19578101, SRR19578102, SRR19578103, SRR19578104, SRR19578105, SRR19578106, SRR19578107, SRR19578113, SRR19578114, SRR20545806, SRR20545807, SRR20545808, SRR20545809, SRR20545814, SRR20545815 at the following link: https://www.ncbi.nlm.nih.gov/Traces/study/?acc=PRJNA844162&o=library_name_s%3Aa.

## Ethics statement

The animal study was reviewed and approved by the Institutional Animal Care and Use Committee of the Ludong University (protocol number LDU-IRB20210308NXY) and the China Government Principles for the Utilization and Care of Invertebrate Animals Used in Testing, Research, and Training (State Science and Technology Commission of the People’s Republic of China for No. 2, October 31, 1988.

## Author contributions

ZL and JY designed and supervised the study. XB, YF, XX, GS and BL prepared the samples. XB, WW, XC, XX and XL analyzed all sequencing data. XB and ZL wrote the manuscript. All authors have read and approved the final manuscript.

## Funding

This research was supported by China Agriculture Research System of MOF and MARA, the National Natural Science Foundation of China (No. 42006077), and the Natural Science Foundation of Shandong Province (No. ZR2019BC052).

## Conflict of interest

The authors declare that the research was conducted in the absence of any commercial or financial relationships that could be construed as a potential conflict of interest.

## Publisher’s note

All claims expressed in this article are solely those of the authors and do not necessarily represent those of their affiliated organizations, or those of the publisher, the editors and the reviewers. Any product that may be evaluated in this article, or claim that may be made by its manufacturer, is not guaranteed or endorsed by the publisher.
